# Mass Diffusion Metamaterials with “Plug and Switch” Modules for Ion Cloaking, Concentrating, and Selection: Design and Experiments

**DOI:** 10.1002/advs.202201032

**Published:** 2022-08-17

**Authors:** Yang Li, Chengye Yu, Chuanbao Liu, Zhengjiao Xu, Yanjing Su, Lijie Qiao, Ji Zhou, Yang Bai

**Affiliations:** ^1^ Beijing Advanced Innovation Center for Materials Genome Engineering Institute for Advanced Materials and Technology University of Science and Technology Beijing Beijing 100083 China; ^2^ School of Materials Science and Engineering University of Science and Technology Beijing Beijing 100083 China; ^3^ State Key Laboratory of New Ceramics and Fine Processing School of Materials Science and Engineering Tsinghua University Beijing 100084 China

**Keywords:** mass diffusion, metamaterials, scattering cancellation

## Abstract

The outstanding abilities of metamaterials to manipulate physical fields are extensively studied in both wave‐based and diffusion‐based fields. However, mass diffusion metamaterials, with the ability to manipulate diffusion with practical applications associated with chemical and biochemical engineering, have not yet been experimentally demonstrated. In this work, ion cloaking, concentrating, and selection in liquid solvents are verified by both simulations and experiments, and the concept of a “plug and switch” metamaterial is proposed based on scattering cancellation (SC) to achieve switchable functions by plugging modularized functional units into a functional motherboard. Plugging in any module barely affects the environmental diffusion field, but the module choice impacts different diffusion behaviors in the central region. Cloaking strictly hinds ion diffusion, and concentrating increase diffusion flux, while cytomembrane‐like ion selection permits the entrance of some ions but blocks others. In addition, these functions are demonstrated in special applications like the catalytic enhancement by the concentrator and the protein protection by the ion selector. This work not only experimentally demonstrates the effective manipulation of mass diffusion by metamaterials, but also shows that the “plug and switch” design is extensible and reconfigurable. It facilitates novel applications including sustained drug release, catalytic enhancement, bioinspired cytomembranes, etc.

## Graphical abstract

Modular mass diffusion metamaterials with reconfigurable functions are designed. The ability to manipulate mass diffusion behavior in a liquid solvent to realize cloaking, concentrating, and selection are experimentally verified. The effective manipulation of mass diffusion behavior by mass diffusion metamaterials can further spawn applications in chemical and biological engineering, such as catalytic enhancement and protein protection.

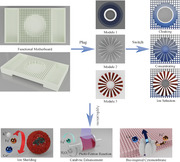

## 1 Introduction

Metamaterials have become the hottest topic in this century because of their exotic electromagnetic properties not found in naturally occurring materials. These properties originate from the outstanding ability of metamaterials to manipulate the physical fields. In particular, the idea of an invisibility cloak, which can render an object invisible to observers, has attracted significant attention. It controls the path of electromagnetic wave transport to achieve invisibility through an inhomogeneous medium artificially designed by the theories of transformation optics^[^ ^1^ ^]^ (TO) by Pendry and SC^[^ ^2^ ^]^ by Engheta. In addition to the ideal cloaking proposed by theory and demonstrated by experiments, the design is further extended to metamaterials with diversified functions such as concentrators,^[^ ^3^, ^4^ ^]^ rotators,^[^ ^5^, ^6^ ^]^ illusion metamaterials,^[^ ^7^, ^8^ ^]^ encryption,^[^ ^9^ ^]^ camouflage,^[^ ^10^, ^11^ ^]^ encoding^[^ ^12^ ^]^ and diode.^[^ ^13^, ^14^ ^]^ After great success in manipulating electromagnetic waves,^[^ ^15^, ^16^, ^17^, ^18^, ^19^, ^20^ ^]^ TO and SC were also proven to be powerful tools to manipulate not only other wave‐based fields like acoustic waves^[^ ^21^, ^22^, ^23^ ^]^ and water waves^[^ ^24^, ^25^, ^26^ ^]^ but also diffusion fields such as thermal flux,^[^ ^27^, ^28^, ^29^, ^30^, ^31^, ^32^, ^33^, ^34^, ^35^, ^36^, ^37^, ^38^, ^39^, ^40^ ^]^ electric fields,^[^ ^41^, ^42^, ^43^, ^44^ ^]^ magnetic fields,^[^ ^45^, ^46^, ^47^, ^48^, ^49^ ^]^ diffusive light^[^ ^50^, ^51^, ^52^ ^]^ and mass diffusion.^[^ ^53^, ^54^, ^55^, ^56^, ^57^, ^58^, ^59^, ^60^, ^61^, ^62^ ^]^ For comparison, metamaterials designed by TO usually exhibit substantial inhomogeneity and anisotropy, while SC provides a simpler way to achieve cloaking for diffusion fields by obeying the Laplacian equation, i.e., Laplacian fields.

As a fundamental phenomenon in nature, mass diffusion occurs spontaneously and depends on the concentration gradient, following Laplace's equation, i.e., a typical Laplacian field. For years, researchers have devoted their efforts to seek a more effective method to control the behavior of diffusion for applications in chemical and biological engineering. Guenneau et al. introduce the idea of manipulating chemical flow by metamaterials and proposed the design of mass diffusion cloaking.^[^ ^53^ ^]^ Soon after, Song et al. experimentally verified the effective cloaking of chloride ions in concrete.^[^ ^54^ ^]^ Maldovan et al. then proposed the concepts of a series of mass diffusion metamaterials that contain functions of cloaking, focusing, and mass separation.^[^ ^57^, ^58^, ^59^, ^60^, ^61^ ^]^ To date, most studies are limited to theoretical design and numerical simulations rather than experiments, and those that do involve experiments deal only with tractable solid samples instead of the more general liquid environments.^[^ ^54^ ^]^ Additionally, the previously proposed mass diffusion metamaterials are out of reconfigurable features. Compared to metamaterials used in electromagnetic fields and thermal fields that have seen massive advances, mass diffusion metamaterials are still prototypes and need great improvements.

**Figure 1:**
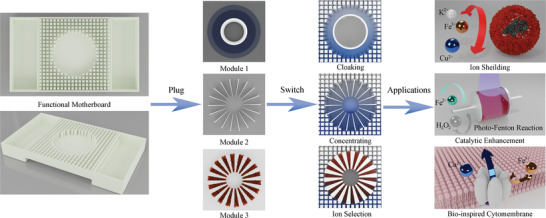
Conceptual illustration of “plug and switch” metamaterial. By Plugging in different modules in the functional motherboard, the mass diffusion metamaterials can manipulate the behavior of ion diffusion to realize ion cloaking, concentrating, and ion selection in liquid solvents. The modularized metamaterials with integrated ion manipulating functions can be further extended to applications like ion shielding, catalytic enhancement, and bio‐inspired cytomembrane.

As metamaterials are different from traditional materials, the key feature of cloaking and concentrating by metamaterials is to realize the minimum and maximum concentration gradient in a certain region while not altering the environmental concentration distribution. This feature is crucial to prevent specific objects from being detected through concentration variation in some chemical and biological applications. To achieve cloaking and concentrating, tailored anisotropic parameters (specifically, the diffusion coefficient) are required, which are proposed to be resolved by various passive and active components. Although the solid host is much easier to model and simulate, the extremely low diffusion efficiency is not appropriate for practical applications of chemical engineering and bioengineering, including ion separation, bioinspired devices, and drug delivery. In this work, we design mass diffusion metamaterials with different functions for manipulating ion diffusion in liquids, including a bilayer cloak based on the SC theory, a concentrator with a fan‐shaped structure, and an ion selector. In addition, we propose the idea of “plug and switch” in a metamaterial device, in which different functions are switchable by plugging modularized functional units into the same motherboard without affecting the environmental concentration, as illustrated in **Figure** **1**. Furthermore, the designed functions are experimentally demonstrated in special applications of catalytic enhancement and protein protection that will benefit catalytic engineering and bioengineering.

## 2 Results

The experimental setup contains a functional motherboard with optimized diffusivity and some pluggable modules to switch the function of metamaterials, as shown in Figure 1. In this work, Modules 1, 2, and 3 represent the cloak, concentrator, and ion selector, respectively. They have identical geometric configurations of a 3 cm annulus with a 1 cm concentric annulus and can be plugged into the center of the same motherboard. The proposed idea of “plug and switch” with modularization design can achieve minimum perturbation to the concentration distribution in the motherboard after plugging in any module and switch the device to functions such as cloaking, concentrating, ion selection, and other possible applications.

### 2.1 Module 1: Bilayer Mass Diffusion Cloak

First, we design a bilayer cloak based on SC theory rather than TO theory to avoid complex anisotropic parameters following a similar method of designing a heat/electric cloak.^[^ ^33^, ^42^ ^]^ A diffusion process without convection can be expressed according to Fick's second law:

(1) $$\frac{\partial C}{\partial t}=\nabla \left(D\nabla C\right)$$

where *t* is time, *C* is the mass concentration (mol m^−3^), *D* is the diffusion coefficient (m^2^ s^−1^), and $\nabla =\frac{\partial }{x}\vec{i}+\frac{\partial }{y}\vec{j}+\frac{\partial }{z}\vec{k}$ is the operator in the Cartesian coordinate system. In a stable state without particle sources, the concentration distribution follows the equation ∇^2^ *C* = 0. For a sphere with a radius of R_1_ enclosed by a bilayer spherical cloak with an inner radius of R_2_ and an outer radius of R_3_, the concentration distribution in this sphere is expressed by:

(2) $$c_{1}=-\sum_{l=1}^{\infty }\left[A_{l}r^{l}+B_{l}r^{-(l+1)}\right]P_{l}\left(\cos\theta \right)\;\left(r\leq R_{1}\right)$$

where *P_l_* (cos *θ*) is a first‐order Legendre polynomial, and *A_l_*, *B_l_* are undetermined constants. Similarly, we have the concentration distribution for the bilayer cloak:

(3) $$\begin{array}{ccc} c_{2} & = & -\sum_{l=1}^{\infty }\left[C_{l}r^{l}+E_{l}r^{-(l+1)}\right]P_{l}\left(\cos\theta \right)\;\left(R_{1}<r\leq R_{2}\right)\\ c_{3} & = & -\sum_{l=1}^{\infty }\left[F_{l}r^{l}+G_{l}r^{-(l+1)}\right]P_{l}\left(\cos\theta \right)\;\left(R_{2}<r\leq R_{3}\right)\\ c_{4} & = & -\sum_{l=1}^{\infty }\left[H_{l}r^{l}+I_{l}r^{-(l+1)}\right]P_{l}\left(\cos\theta \right)\;\left(r>R_{3}\right) \end{array}$$

If *r* → ∞, then *c*_4_= − c_0_ *rcosθ*. We therefore only have to consider *l* = 1; if *r* → 0, then *c*_1_ is limited, then *B*_1_=0. Also, we have continuous conditions at boundaries:

(4) $$\begin{array}{c} {\left.c_{i}\right|}_{r=R_{1},R_{2},R_{3}}\;=\;{\left.c_{i+1}\right|}_{r=R_{1},R_{2},R_{3}},{\left.D_{i}\frac{\partial c_{i}}{\partial r}\right|}_{r=R_{1},R_{2},R_{3}}\;={\left.D_{i+1}\frac{\partial c_{i+1}}{\partial r}\right|}_{r=R_{1},R_{2},R_{3}} \end{array}$$

where *D_i_* refer to the diffusivity of the central region, inner layer, outer layer of bilayer cloak, and background medium when i increasing from 1 to 4. By setting *D*_2_ = 0 (diffusivity of the inner layer), the region inside the cloak can keep a uniform concentration (*c*_1_ is limited). Then, we only have to deal with the concentration distribution outside the cloak. By substituting Equation (1) and (2) into Equation (3), we have:

(5) $$H_{1}=c_{0}R_{2}^{3}\frac{D_{3}\left(2R_{2}^{3}-2R_{1}^{3}\right)-D_{4}\left(2R_{2}^{3}+R_{1}^{3}\right)}{D_{3}\left(2R_{2}^{3}-2R_{1}^{3}\right)+2D_{4}\left(2R_{2}^{3}+R_{1}^{3}\right)}$$

By setting *H*_1_ = 0, we have parameters for the bilayer cloak:

(6) $$D=\frac{2R_{2}^{3}+R_{1}^{3}}{2\left(R_{2}^{3}-R_{1}^{3}\right)}D_{0}$$

For the 2D case (corresponding to the bilayer annulus):

(7) $$D=\frac{R_{2}^{2}+R_{1}^{2}}{R_{2}^{2}-R_{1}^{2}}D_{0}$$

where *D* is the diffusion coefficient of the outer layer of the bilayer cloak and *D*_0_ is the diffusion coefficient of the background medium.

According to the above derivation process, a bilayer cloak requires an inner layer with an index of zero and a complementary outer layer with an index associated with the background media according to Equation (4) or (5).

For a 2D mass diffusion cloak, we use a 3D printed resin annulus (functional unit 1) to realize the zero index, which leaves only the diffusivities of the outer layer of the cloak and the background media to be engineered. Then, we use the effective medium theory (EMT) to match the diffusivity of the outer layer of the cloak with that of the background media. The radii of the inner layer and the outer layer are set to 2 and 3 cm, respectively. The outer layer is pure water, and the internal layer is a 2 mm annulus made of resin. Therefore, the diffusivity ratio of the outer layer of the cloak and the background media is calculated as 13/5 according to Equation (5). Similar to the calculations in thermal physics,^[^ ^32^, ^63^, ^64^ ^]^ we expand the Bruggeman approximation of EMT to acquire specific diffusivity. For a two‐phase system composed of water and homogeneously distributed resin pillars, the effective diffusivity is obtained as:

(8) $$\begin{array}{ccc} & & f_{1}\frac{D_{1}-D_{e}}{2D_{e}+D_{1}}+f_{2}\frac{D_{2}-D_{e}}{2D_{e}+D_{2}}=0\\ & & f_{1}+f_{2}=1 \end{array}$$

where *f*_1_ and *f*_2_ are the volume ratios of water and resin pillars, *D*_1_ and *D*_2_ are the diffusivities of water and resin pillars and *D*_e_ is the effective diffusivity of the two‐phase system. The calculated *f*_2_ is 16/39. The difference in ion diffusivity of the two phases is on the scale of several orders of magnitude, so the ion diffusivity in resin pillars can be considered zero when the filling ratio of resin is small. In addition, we evaluate the dependence of the effective diffusivity on the filling ratio, as shown in S Figure S1a, (Supporting Information). The approximation holds true when the effective diffusivity is high, while the effective diffusivity is linearly related to the filling ratio. However, if the effective diffusivity is close to zero, the ion diffusivity in resin pillars cannot be neglected. The optimal parameters for the functional motherboard are shown in Figure S1b, (Supporting Information).

**Figure 2:**
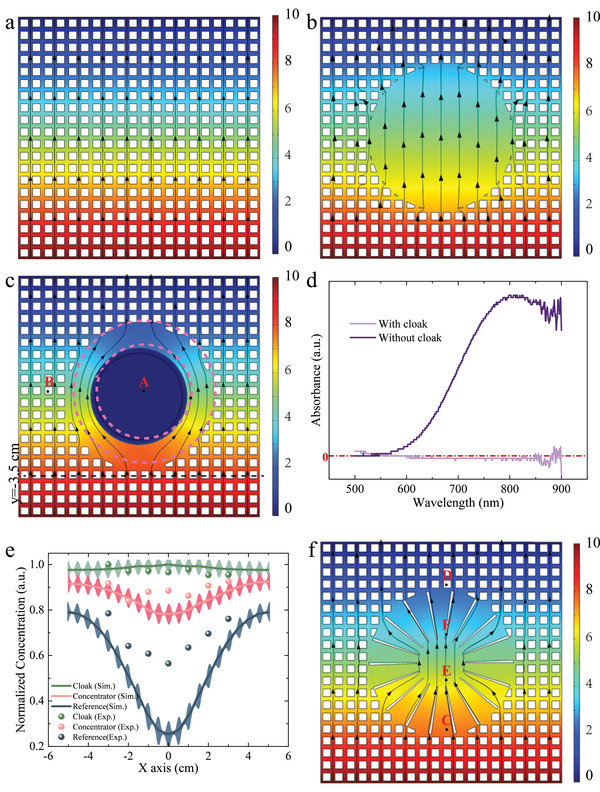
Simulated and measured results of cloak and concentrator. a) Simulated concentration surface and flux lines for the background. b) Simulated concentration surface and flux lines for reference. c) Simulated concentration surface and flux lines for Cloak. d) Measure concentration at point A with and without a cloak. e) Measure results of concentration distribution at line y = −3.5 for reference, cloak, and concentrator. Error bars are plotted based on Figure S5, (Supporting Information) to evaluate the scattering of resin pillars. f) Simulated concentration surface and flux lines for the concentrator.

The concentration distribution for the metamaterial device is simulated and shown in **Figure** **2**. The concentration distribution exhibits straight streamlines in a homogeneous medium with uniformly distributed resin pillars in the water tank, as shown in Figure 2a. However, it is clearly scattered when a circular area is removed, as shown in Figure 2b. The undisturbed concentration distribution outside the cloak is restored when the cloaking module is plugged in and the concentration inside the cloak is nearly zero, as shown in Figure 2c. The chemical flux is guided around the central region in the cloak and maintains a uniform distribution outside the cloak. This behavior indicates that the central region is chemically invisible to observers and that ion shielding is achieved. We also compare the ideal cloak of homogeneous diffusivity with that of inhomogeneous diffusivity designed by EMT through simulation, as shown in Figure S2, (Supporting Information). To characterize the concentration contour, we measured the concentration distribution along a line y = −3.5 cm close to the cloak, which is shown in Figure 2c. The concentration distributions at line y = −3.5 cm in these two scenarios are close, while the error is mainly because the equivalent parameters of EMT require random distribution of micro/nanoparticles in the two‐phase system.

To experimentally verify the cloaking effect, we selected the CuSO_4_ solution as an example due to its distinguishing color and added the CuSO_4_ solution and deionized water to the left and right tanks in the metamaterial device. After waiting 5 min for the solution to reach a steady state, we took samples of the solution from points inside (A) and outside (B) the cloak and along a line behind the cloak and measured the concentrations by a spectrophotometer. The concentration ***c*** is characterized by absorbance as follows:

(9) $$A\left(\lambda \right)=\alpha \left(\lambda \right)\cdot c\cdot l$$

where A(*λ*) is the absorbance, *α*(*λ*) is the absorption coefficient and *l* is the thickness of the sample. Since *α*(*λ*) and *l* are constants for a given solution with the same solute, the concentration is linearly related to the measured absorbance. Note that the mixed system we choose consists of CuSO_4_/K_2_SO_4_, in which K^+^ has no obvious absorption peaks that can be measured directly by spectrophotometry, so the measured absorption peaks are those of Cu^2+^ and can be used to characterize the magnitude of copper ion concentration. Otherwise, if the ions of the mixed system have overlapping absorption peaks, then Lambert‐Beer law will no longer apply. The measured absorbances of points A and B are shown in Figure 2d. The concentration of the cupric cation inside the cloak is found to be zero; this region inside the cloak benefits from the isolated conditions provided by Module 1. In addition, we take 300 µL of solution from 7 evenly spaced spots along the line at y = −3.5 cm and measure the absorbance, as shown in Figure S3, (Supporting Information). The measured results indicate that the scattering of the chemical gradient is drastically reduced, similar to the simulated results, as shown in Figure 2e. That is, chemical cloaking is achieved. The detailed results of the absorbance measurements are shown in Figure S4, (Supporting Information). The measured results may represent the average concentration around the pipette because of deviations in the sampling point location. To determine the possible error, we also simulate the concentration distribution astride the line y = −3.5 cm, i.e., along line y = −3.45 cm and y = −3.55 cm, as shown in Figure S5, (Supporting Information). We notice that the concentration distribution has intense scattering when close to resin pillars, which may cause test errors during the sampling of the solution. The perturbations of concentration along lines y = −3.45 cm and y = −3.55 cm cancel each other out on average.

### 2.2 Module 2: Fan‐Shaped Mass Diffusion Concentrator

Second, an ion concentrator is designed in a typical fan structure as Module 2, where the invisibility is achieved by minimum perturbation when ${D^{\prime }}_{r}{D^{\prime }}_{\theta }=D_{0}^{2}$. Although previous works have suggested that a mass diffusion concentrator can be designed using TO,^[^ ^58^, ^59^ ^]^ here we follow the solutions in the thermal concentrator^[^ ^4^ ^]^ by setting: *D*′_*r*_=2^*n*^ *D*_0_,*D*′_*θ*_ = 2^−*n*^ *D*_0_. To create a concentrator, *D*′_*r*_ ≫ *D*′_*θ*_ is required, which indicates that the general ion diffusion is towards the radial direction and that the anisotropy determines the concentration efficiency $\frac{C_{E}-C_{F}}{C_{C}-C_{D}}$. Based on the EMT *D*′_*r*_=*D*_A_ *f_A_* +*D*_B_ *f_B_*,*D*′_*θ*_ = 1/(*f_A_*/*D*_A_ + *f_B_*/*D*_B_), we chose water and resin as materials A and B, respectively. Then, the exact integration of cloaking and concentrator in the modularized metamaterials should fulfill the following condition:

(10) $$D=\frac{R_{2}^{2}+R_{1}^{2}}{R_{2}^{2}-R_{1}^{2}}\sqrt{{D^{\prime }}_{r}{D^{\prime }}_{\theta }}$$

where *D* is the diffusion coefficient of the outer layer of the bilayer cloak (Module 1), *D*′_*r*_ and *D*′_*θ*_ is the radical and tangential mass diffusion coefficient of the concentrator (Module 2). Equation (10) illustrates well the conditions for the integration of the cloak and the concentrator.

**Figure 3:**
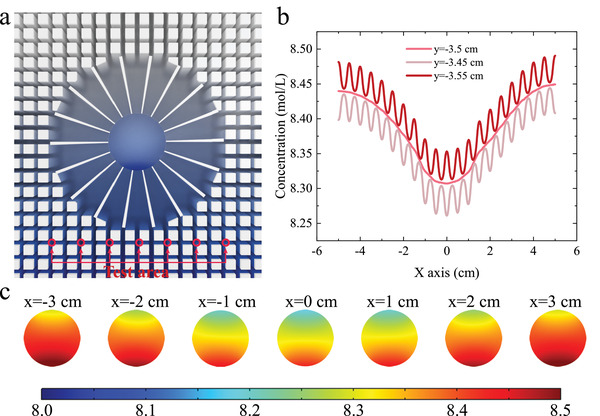
Measurement error analysis. a) Schematic illustration of the seven test points. b) Simulated results of concentration distribution of the concentrator at line y = −3.5, −3.45, −3.55 cm. c) Simulated results of the concentration distribution of the concentrator at the seven test points.

As an intractable point of this design, an accurate measure of the diffusivity of cupric cations in resin is unknown and is also affected by many factors, including concentration and temperature. These factors make it difficult to match the diffusivity to satisfy ${D^{\prime }}_{r}{D^{\prime }}_{\theta }=D_{0}^{2}$. In order to minimize the disturbance of the external concentration distribution by the concentrator, we need to increase the diffusion coefficient in the tangential direction. Therefore, the Concentrator needs to have as small a percentage of resin as possible while the effective medium theory holds. However, due to the limitations of 3D printing technology, the resin is too thin near the central region which will reduce the strength of the structure, so the final resin volume ratio was optimized to 10%. Then, the diffusivity of *D*′_*r*_ decreases to ≈80% of that in pure water and *D*′_*θ*_ to 0. The simulated concentration distribution for the metamaterial device with the concentrator module demonstrates that although the parameters do not strictly obey the formula, the scattering is significantly reduced compared to the blank reference, and a significantly concentrated gradient is developed in the central region, as shown in Figure 2f. The measured concentration along the line at y = −3.5 cm in Figure 2e is almost identical to the simulated results. However, note that the data of the concentrator in the middle did not exactly agree with the simulated data, which is partially due to experimental errors, such as leakage of the liquid at the boundary. The measured test points are illustrated in **Figure** **3**a. It can be found that although we want to accurately test the concentration at the point when a pipette is used to aspirate a solution, it tends to aspirate an area of the solution. Therefore, the error may also originate from the uniform distribution of resin pillars in the background medium. Although the diffusion coefficients of the background medium acquired by the effective medium theory are reliable, mass does not diffuse in it as they do in a homogeneous medium. As Figure 3b shows, the concentration distribution fluctuates sharply close to the resin pillars, and the fact that the concentration testing process requires aspirating the solution in the gap between the pillars inevitably introduces errors. To better illustrate the actually tested concentration of the solution at the point, we simulated the concentration distribution of the area around each point, as shown in Figure 3c. It can be noticed that the concentration distribution over an entire region is not homogeneous, and although the average concentration over the entire region is comparable to the actual concentration at the point, it still introduces a certain amount of error, which is a challenge that needs to be overcome for mass diffusion metamaterials.

We then extend the functions of cloaking and concentrating to the catalytic enhancement and protein protection by experimental demonstration. The concentrating feature of Module 2 significantly increases the concentration gradient in the central region, which can be used to promote catalytic efficiency. We select the typical Fenton reagent as an example, which is widely used in chemical engineering to remove refractory organic pollutants. As a strong oxidation system composed of Fe^2+^ and H_2_O_2_, the Fenton reagent generates hydroxyl radicals and superoxide radicals with extremely strong oxidization through a chain reaction:

(11) $$\begin{array}{c} {\mathrm{Fe}}^{2+}+H_{2}O_{2}\to {\mathrm{Fe}}^{3+}+\mathrm{HO}\cdot +{\mathrm{OH}}^{-}\\ {\mathrm{Fe}}^{3+}+H_{2}O_{2}\to {\mathrm{Fe}}^{2+}+\mathrm{HO}O\cdot +H^{+}\\ 2H_{2}O_{2}\to \mathrm{HO}\cdot +\mathrm{HOO}\cdot +H_{2}O \end{array}$$

**Figure 4:**
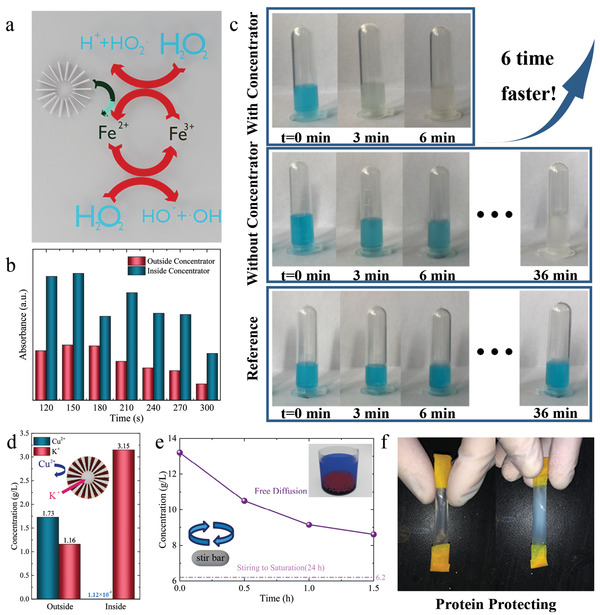
Demonstration of catalytic and bioengineering applications. a) Illustration of Fenton reaction boosted by the concentrator. b) Time‐dependent measurement of concentration inside and outside the concentrator. c) Observation of catalytic boost by the concentrator. d) Concentration inside and outside the ion selecting metamaterials in concentration gradient of CuSO_4_/K_2_SO_4_. e) Adsorption of cupric cation by ion exchange resin. f) Demonstration of protein protecting application by ion selecting metamaterials.

Although the mechanism of the above redox cycle reaction is unclear due to the complicated intermediate products, most researchers have suggested that hydroxyl groups are the origin of intense oxidation of the Fenton reagent and that Fe^2+^ serves as a catalyst, as shown in **Figure** **4**a. Here, the above chain reaction is simply treated as the disproportionation of hydrogen peroxide. We plug Module 2 into the motherboard and inject FeSO_4_ solution and deionized water into the two tanks. The concentrations in the central region with and without Module 2 are measured, and a 150% increase in the concentration of Fe^2+^ is found at *t* = 120 s. We take the solution inside and outside the concentrator every 30 s to evaluate the concentration change with time, as shown in Figure 4b. At every measured timepoint, the concentration inside the concentrator is obviously higher than that outside. The reduction in concentration outside of the concentrator is because the solution concentration is more susceptible to being disturbed by sampling as it approaches equilibrium.

To evaluate the catalytic efficiency of the Fenton reaction in dye degradation, we add the Fe^2+^ solution taken from inside the concentrator into the precursor solution of Fenton reagent with organic dye (Specimen 1) and add the solution from outside the concentrator to another precursor solution (Specimen 2). In addition, Specimen 3 was made by adding the deionized water to the precursor solution as a blank control. The comparison of the degradation effect for different specimens is shown in Figure 4c, and all of the results are shown in Figure S6, (Supporting Information). The blank control indicates that organic dye cannot be degraded by pure water. The organic dye is completely degraded after 6 min in Specimen 1, while it takes ≈36 min to fully degrade in Specimen 2. This result indicates that the catalytic efficiency is 6 times higher when the metamaterial device is used. Note that the concentration only has a slight concentration enhancement compared to that outside the concentrator. However, the experimental demonstration in Figure 4c shows that the reaction time is 5 times less in the presence of the concentrator module. This is due to the insufficient amount of Fe^2+^ involved in the chain reaction of Fenton's reagent, which gradually decreases in concentration as the reaction proceeds leading to a reduction in the catalytic rate. It can be expected that if the concentration of Fe^2+^ is sufficient, the difference in catalytic rate between the two sets of experiments will not be so large. The experimental results prove that the concentrator greatly promotes catalytic applications, which can be further extended to built‐in metamaterials for catalytic enhancement.

### 2.3 Module 3: Mass Diffusion Selector

The previously discussed cloak and concentrator modules remarkably change the field distribution in the central region but do not disturb the surrounding field, so they do not destroy the environmental field distribution and avoid detection through field fluctuations when they work. However, the process becomes much more complex for mass diffusion metamaterials because diffusion may involve a variety of ions. This is a problem not faced by electromagnetic or other metamaterials. Similar to leukocytes, which detect viruses or bacteria through antigenic determinants, mass diffusion metamaterials have the important ability to screen and selectively manipulate different ions. Here, we design the third module for the mass separation function as suggested in previous work. The third module functions as a bioinspired cytomembrane‐like ion selector. It enables specific ion penetration or shielding and maintains acid‐base equilibrium.

Module 3 has a fan structure filled with an ion exchange resin. The ion‐exchange resin contains sulfonic acid groups, carboxyl groups, and phenol groups that exchange with high valent cations. As a result, the high‐valent cations of Cu^2+^ are shielded out of the central region, but low‐valent ions such as K^+^ and Na^+^ are still permitted. We demonstrate the design of a potassium ion channel. When the metamaterial device with an ion selection module is in the CuSO_4_/K_2_SO_4_ gradient, the cation exchange resin preferentially reacts with cupric cations and allows potassium ions to achieve the separation of CuSO_4_/K_2_SO_4_. The measured concentration inside and outside the module at 120 s is shown in Figure 4d. The ion selector limits nearly all cupric cations while promoting the concentration of potassium ions. The concentration distribution along line y = −3.5 cm is also measured and shown in Figure S7, (Supporting Information). The overall concentration is lower than that of the cloak, concentrator, and reference since the ion exchange resin absorbs major cupric cations.

As shown in Figure 4e, when 100 g ion‐exchange resin was mixed with 200 mL of 13.2 g L^−1^ CuSO_4_ solution, the concentration approached a saturation of 8.6 g L^−1^ after 2 h. This result indicates that the ion exchange resin acts as the active component to absorb the cupric cation. In addition, the absorption speed is fast enough to shield the cupric cation in the CuSO_4_ gradient. Since a certain cation is selectively shielded, the metamaterial device with Module 3 can be used as a cytomembrane‐like ion selector to prevent chemical harm in bioengineering. Protein protection is selected as a special application to demonstrate the function in the experiment. As shown in Figure 4f, a dialysis bag sealed with bovine serum albumin (BSA) solution was placed in the center of the motherboard in a CuSO_4_ gradient. BSA has a molecular weight of 66.5 kDa, while that of the dialysis bag is 12 kDa, which means that CuSO_4_ can penetrate the dialysis bag and cause the denaturation of BSA. If Module 3 is absent, BSA is exposed to CuSO_4_ and aggregates because of the conformational change and the loss of solubility. If Module 3 is plugged in, the BSA solution is unaltered after the same amount of time, demonstrating an excellent protein protection performance. Moreover, Module 3 can also be applied to anion shielding by filling the apparatus with anion exchange resin. Both our proposed Fenton reagent catalytic enhancement and protein protection applications are proposed in the context of different ion manipulation functions. That is, manipulation of the gradient properties inside a metamaterial by structural design without changing the external gradient distribution within a uniform gradient field. We can then use the centrally enhanced ion concentration for catalytic enhancement. Alternatively, we use the effect of the center shielding the heavy metal ions to protect the proteins in the central region. These proposed application scenarios are based on the design of metamaterials to manipulate the distribution of ions under a specific condition.

Here, we provide experimental evidence for mass separation. However, the mass separation in this work does not rely on the rational design of the diffusion coefficient, but originates from the selective adsorption of ions by the active component, the ion exchange resin. Also, the ion exchange resin will respond differently depending on the type of ions (valence of ions) in the solution. For example, a system with Cu^2+^ (divalent cation) and K^+^ (monovalent cation) will allow K^+^ to pass while shielding Cu^2+^. A system with the presence of Fe^3+^ and Cu^2+^ will allow Cu^2+^ to pass while shielding Fe^3+^. In addition, adding anionic exchange resin to the resin framework will enable the screening of anions. Thus, although this device is functionally identical to the previous work, the principles used to achieve this function are different.

The metamaterials designed for ion cloaking, concentrating, and selection in this paper are achieved by controlling the spatial distribution of diffusion coefficient which is designed according to effective medium theory. The similar geometry of Module 1/2/3 allows them to be integrated into one motherboard. Module 1 has good invisibility with equivalent parameters in accordance with Equation (7). Modules 2 and 3 are very similar in architecture in that they are both fan‐arranged structures consisting of two media. Module 2 and Module 3 differ in component composition and function. In terms of component composition, Module 2 consists of water and resin, while Module 3 consists of resin, water, and ion exchange resin. In terms of function, Module 2 concentrates all ions in the central region without selectivity; meanwhile, Module 3 screens the valence of the ions (depending on the type of ion exchange resin) that is concentrated in the central region.

Note that although Module 1 of the cloak can also accomplish ion shielding, the strategy is quite different. The ion selector is an open system that selectively blocks some specific ions so that it provides more flexibility in complicated scenarios. Additionally, the ion selection function is limited by the saturation of the cation exchange resin. As more cation exchange resin is used, the length of time that the high‐valent cations are blocked increases, but the effective diffusivity along the radial direction is smaller for other chemicals. This is because the ion exchange resin does not contribute to the diffusion of chemicals. It is suggested that other non‐metallic mineral adsorbents, such as activated carbon, can also achieve ion shielding. Typically, these adsorbents absorb unselectively which is more properly used in water treatment. However, our proposed ion‐exchange resin‐based selector can select certain ions in the mixed ion systems based on the valence state.

The switchable function of Module 1/2/3 is not dynamic; instead, the modules are placed in advance before each diffusion process takes place according to the desired ion diffusion manipulation function.

Module 1/2 can manipulate the diffusion process of single or mixed ion systems without selectivity and are theoretically suitable for most ion systems that do not destroy the resin structure. In contrast, the ion selection function of Module 3 is related to the type of ion exchange resin used and the ionic valence composition of the mixed system. More precisely, the selectivity is more pronounced when there is a large valence difference between the mixed ion systems, and less so when the opposite is true.

## 3 Discussion

In this paper, we proposed a design paradigm for a “plug and switch” metamaterial with pluggable functional units and switchable functions. The simulated and measured results indicate that the cloak can almost entirely reduce scattering, while the concentrator can greatly promote diffusive flux. The results prove that cloaking, concentrating, and ion selection can be experimentally realized in an integrated functional motherboard with modularized functional units. The demonstrated “plug and switch” metamaterials are powerful for manipulating mass diffusion behavior and facilitate novel applications with multiple extensible functions including sustained drug release, catalytic enhancement, bioinspired cytomembranes, etc. The concentrator can greatly enhance the catalytic efficiency by promoting the concentration of catalysis, while the ion selector provides a protein protection case by prohibiting heavy metal ions; this ion selector can be considered a bioinspired cytomembrane with ion channels. The ability of bioinspired cytomembranes to open gives them the potential for use in bioengineering applications such as sustained drug release and artificial organs.

## 4 Experimental Section

### Fabrication of Mass Diffusion Metamaterial

The functional motherboard and functional units were fabricated by LCD‐based stereo lithography appearance 3D printing. The material was UV curable resin. The detailed size parameters were calculated based on effective medium theory. Each functional unit was attached to the motherboard by waterproof glue to prevent the ion in solution from penetrating the cloaked region through the small gap in the bottom.

### Experimental Setup of Cloak and Concentrator

The experimental equipment had two tanks at the left and right end with CuSO_4_ solution and deionized water respectively to produce a certain concentration gradient, and then a diffusion platform with engineered diffusivity was connected to both tanks through a port sealed by a dialysis membrane, which was adopted to slow the diffusion at the port and prevent convection. After the liquid was added to both tanks and the diffusion platform between the tanks, the dialysis membrane was removed slowly.

### Concentration Measurement

Three hundred microliter solution was taken at each spot along line x = −4 cm by a multi‐channel pipette and transferred to a labeled centrifuge tube. The solution was diluted with 3 ml water and measured the absorbance by a spectrophotometer. For the concentration measurement by inductively coupled plasma, the dilution was selective depending on the concentration.

### Preparation of Precursor in Catalytic Boost Demonstration

After 40 mL methyl blue with a concentration of 10 mg L^−1^ was taken and the pH was tuned to ≈3 by H_2_SO_4_, 2 ml H_2_O_2_ was added to the solution and mixed by magnetic stirring to prepare the precursor solution. For the cases with and without a concentrator, 300 µL solution in the central region was taken and mixed with specimens 1 and 2 with 50 ml precursor solution respectively, while specimen 3 adopted 300 µL water as blank control.

### Experimental Setup and Measurement Methods of Catalytic Enhancement

Module 2 was plugged into the motherboard and FeSO_4_ solution and deionized water was injected into the two tanks. The Fe^2+^ solution taken from inside and outside the concentrator was added into the precursor solution of Fenton reagent with organic dye separately. The deionized water was added to the precursor solution as a blank control. The mixed solutions were fully reacted to using magnetic stirring. Every 3 min, the solutions were taken from the 3 reaction systems and immediately photographed to determine the degradation by color.

### Experimental Setup and Measurement Methods of Protein Protection

The BSA solution was prepared. The solution was injected into the dialysis membrane and sealed using waterproof tape. The sealed BSA solution was placed in the middle of the ion selector and the reference respectively, in contact with the solution. 10 min later, the BSA solution was observed for denaturation.

## Conflict of Interest

The authors declare no conflict of interest.

## Author Contributions

Y.L. and Y.B. conceptualized the initial ideas and carried out simulations. Y.L. and Y.B. performed the experiments with support from C.Y and Z.X. All authors were involved in data interpretation. Y.L. prepared the initial manuscript with contributions from all the authors and Y.B. revised the manuscript.

## Supporting information

Supporting Information

## Data Availability

The data that support the findings of this study are available from the corresponding author upon reasonable request.
