# Supplementary material for: Mass Diffusion Metamaterials with “Plug and Switch” Modules for Ion Cloaking, Concentrating, and Selection: Design and Experiments
Source: Adv Sci (Weinh). 2022 Aug 17;9(30):2201032. doi: 10.1002/advs.202201032 (PMC9596857; doi:10.1002/advs.202201032)
Supplement: Supplementary file 1 — Supporting Information [file ADVS-9-2201032-s001.pdf]

ADVANCED SCIENCE
Open Access

Supporting Information

for *Adv. Sci.*, DOI 10.1002/advs.202201032

Mass Diffusion Metamaterials with “Plug and Switch” Modules for Ion Cloaking, Concentrating, and Selection: Design and Experiments

*Yang Li, Chengye Yu, Chuanbao Liu, Zhengjiao Xu, Yanjing Su, Lijie Qiao, Ji Zhou and Yang Bai**

Supporting Information

Mass Diffusion Metamaterials with "Plug and Switch" Modules for Ion Cloaking, Concentrating and Selection: Design and Experiments

Yang Li, Chengye Yu, Chuanbao Liu, Zhengjiao Xu, Yanjing Su, Lijie Qiao, Ji Zhou, and Yang Bai[*]

Y. Li, C. Yu, Z. Xu, Prof. Y. Su, Prof. L. Qiao, Prof. Y. Bai
Beijing Advanced Innovation Center for Materials Genome Engineering
Institute for Advanced Materials and Technology
University of Science and Technology Beijing
Beijing 100083, China
E-mail: baiy@mater.ustb.edu.cn (Y. Bai)

Dr. C. Liu
School of Materials Science and Engineering
University of Science and Technology Beijing
Beijing 100083, China

Prof. J. Zhou
State Key Laboratory of New Ceramics and Fine Processing
School of Materials Science and Engineering
Tsinghua University
Beijing 100084, China

Correspondence and requests for materials should be addressed to Y.B.

Supporting Figure

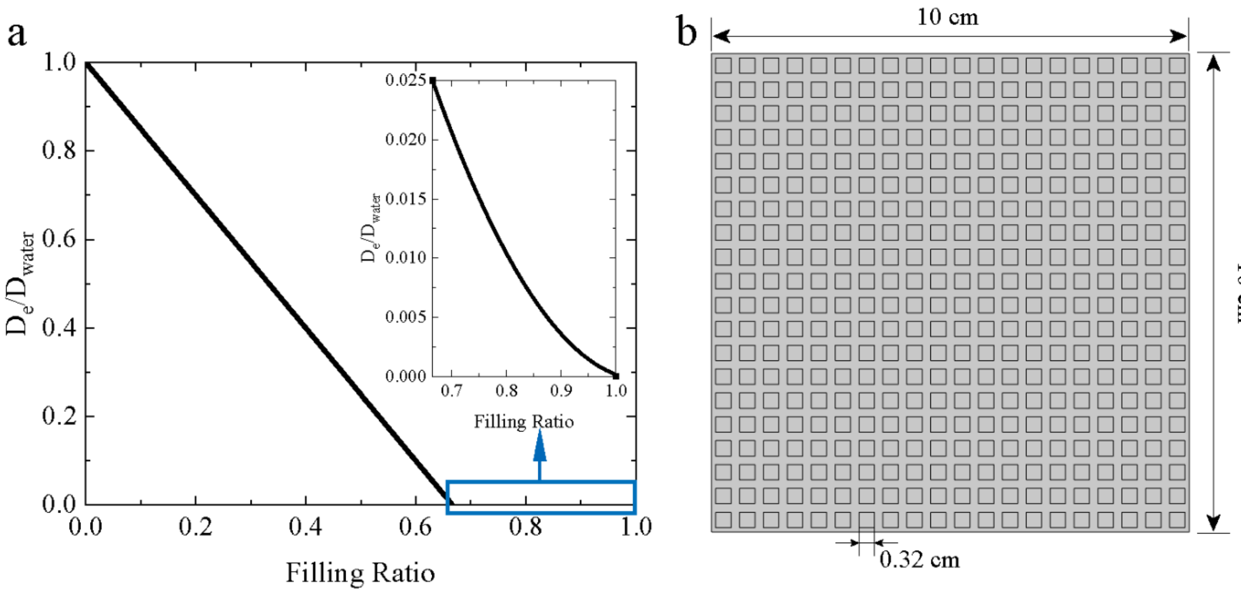

Figure S1. Optimized parameters for effective diffusivity. a Calculated dependence of effective diffusivity on filling ratio of resin pillars. **b** Optimized parameters of background with effective diffusivity of 5/13 D_{water}.

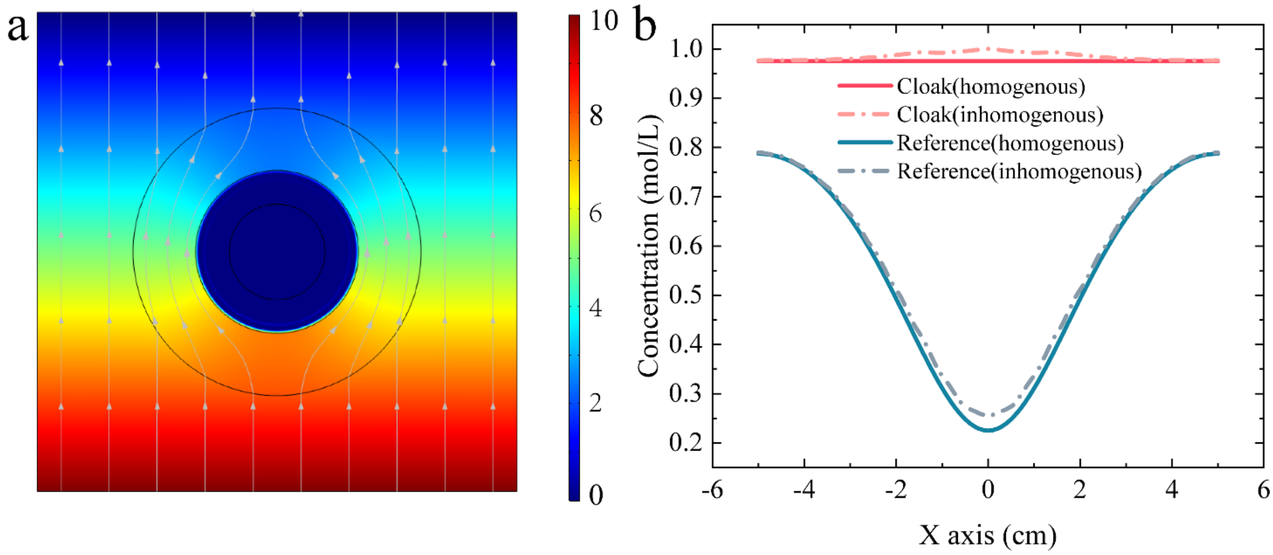

Figure S2. Comparison of cloak with ideal homogenous diffusivity and inhomogeneous diffusivity. a Concentration surface and flux lines for idea cloak. **b** Concentration distribution at line y=-4 cm with and without cloak for homogenous and inhomogeneous cases.

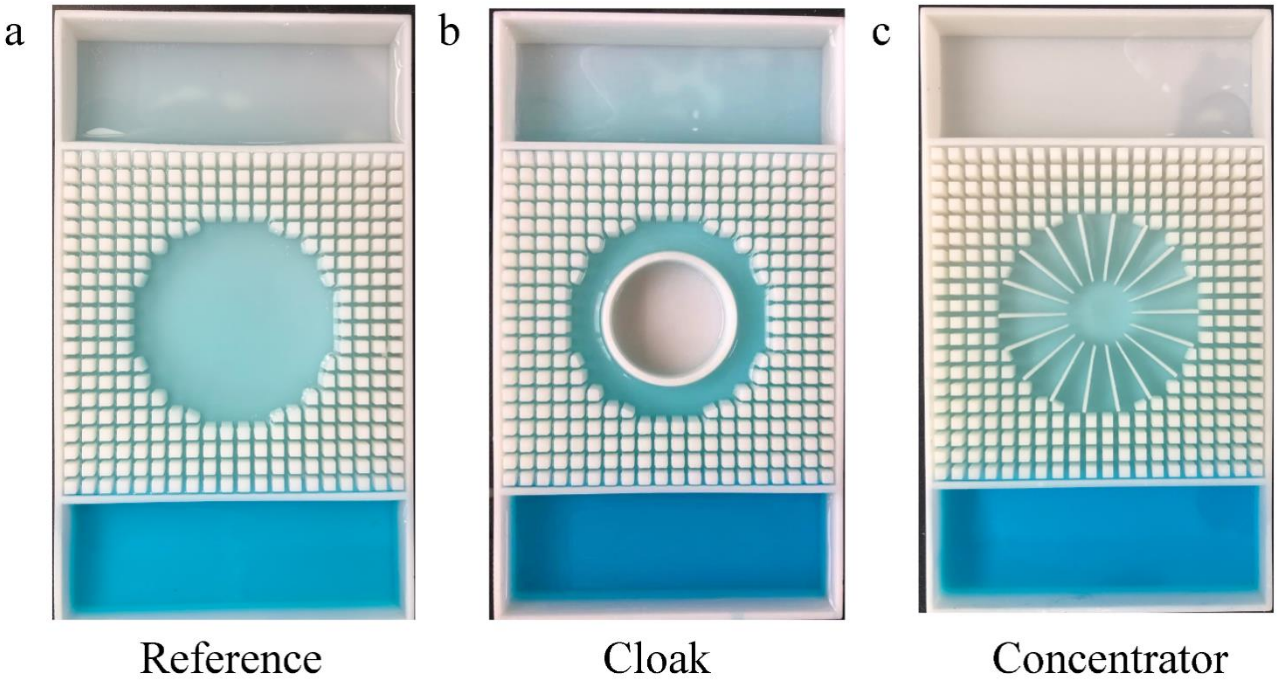

Figure S3. Experimental setup. Multichannel pipette is used to transfer solution along line y= -3.5 cm for **a** reference, **b** cloak and **c** concentrator.

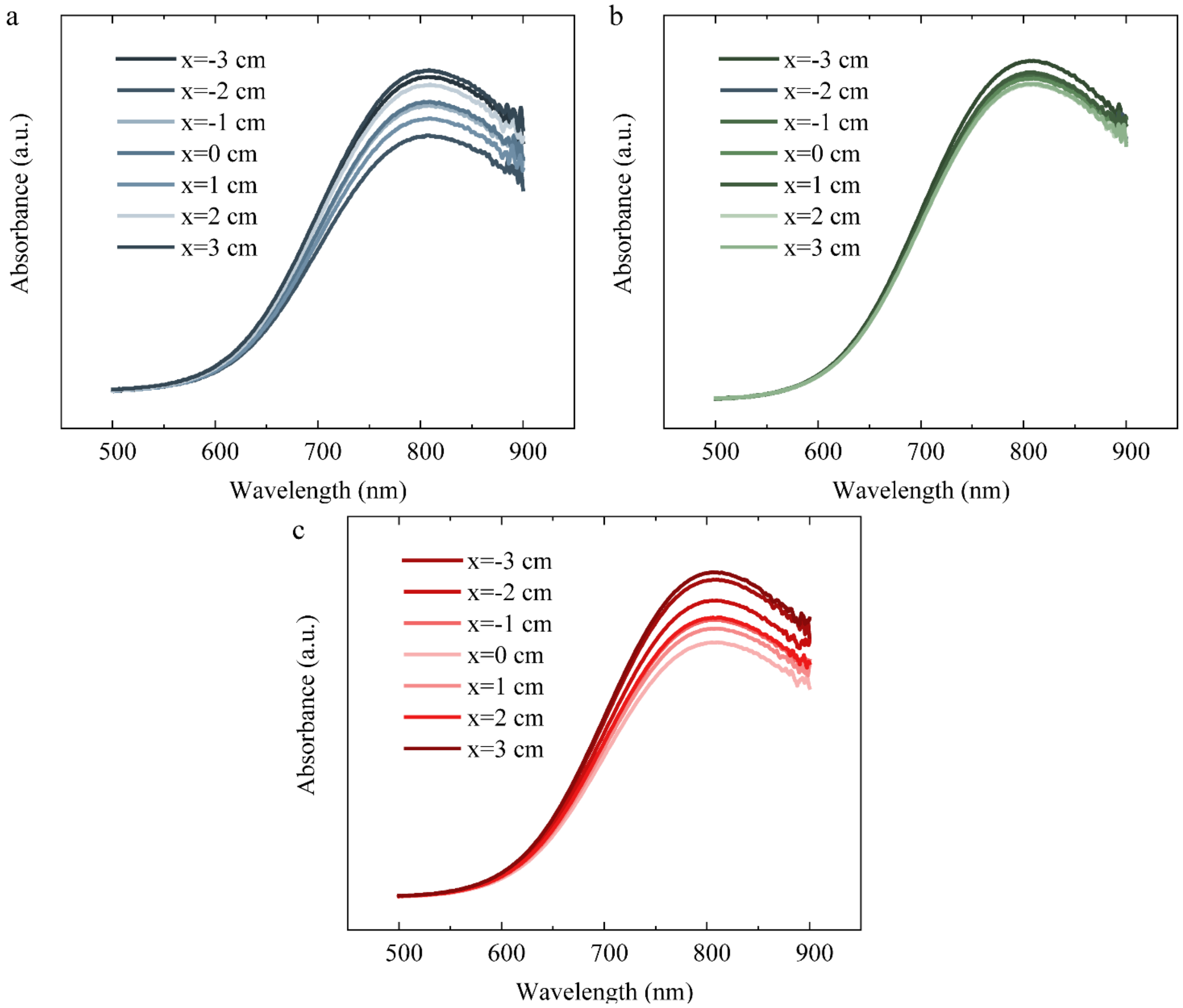

Figure S4. Measured absorbance at line y= -3.5 cm. Absorbance is tested for 7 spots spaced 1 cm for **a** reference, **b** cloak and **c** concentrator.

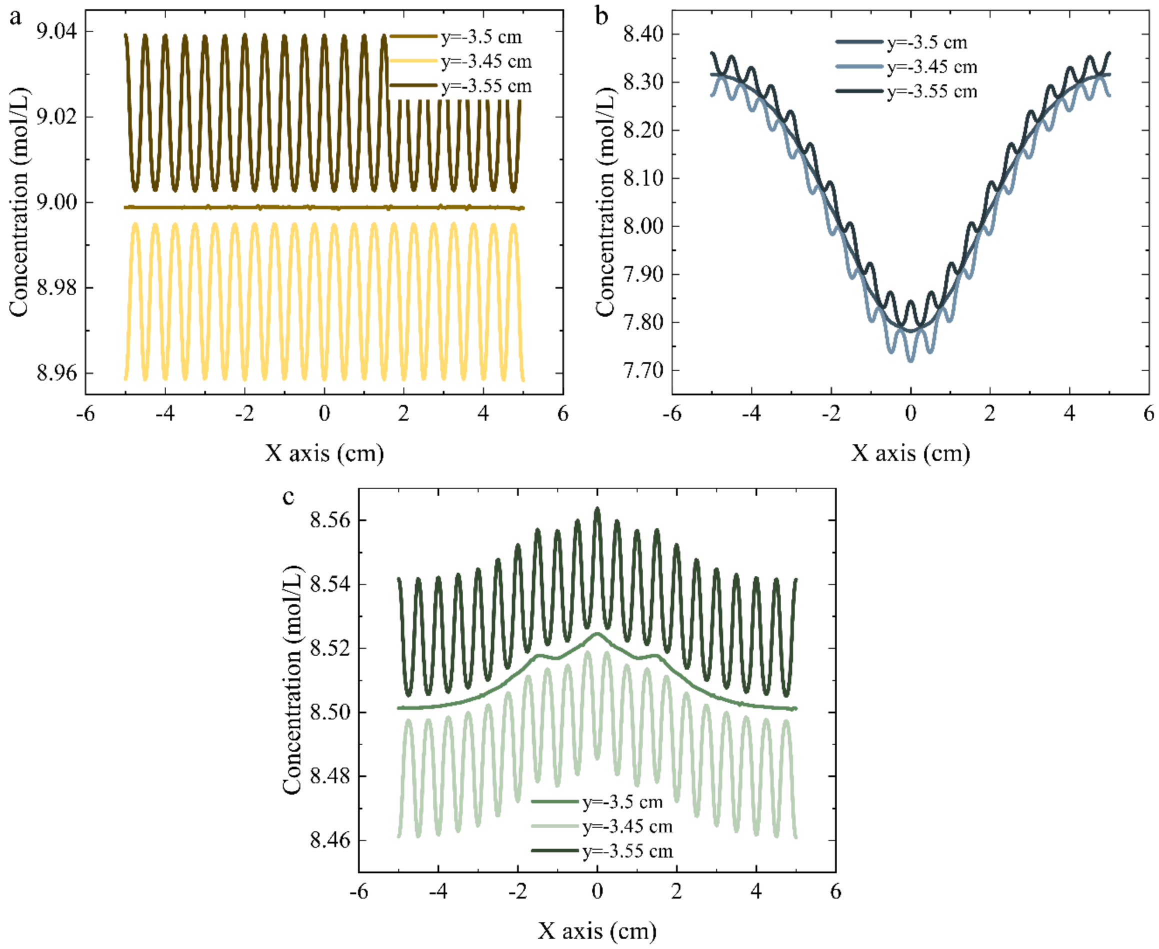

Figure S5. Simulated results of scattering details of resin pillars. Simulated results of concentration distribution at line y= -3.5, -3.45, -3.55 cm. Concentration distribution for **a** background, **b** reference and **c** cloak.

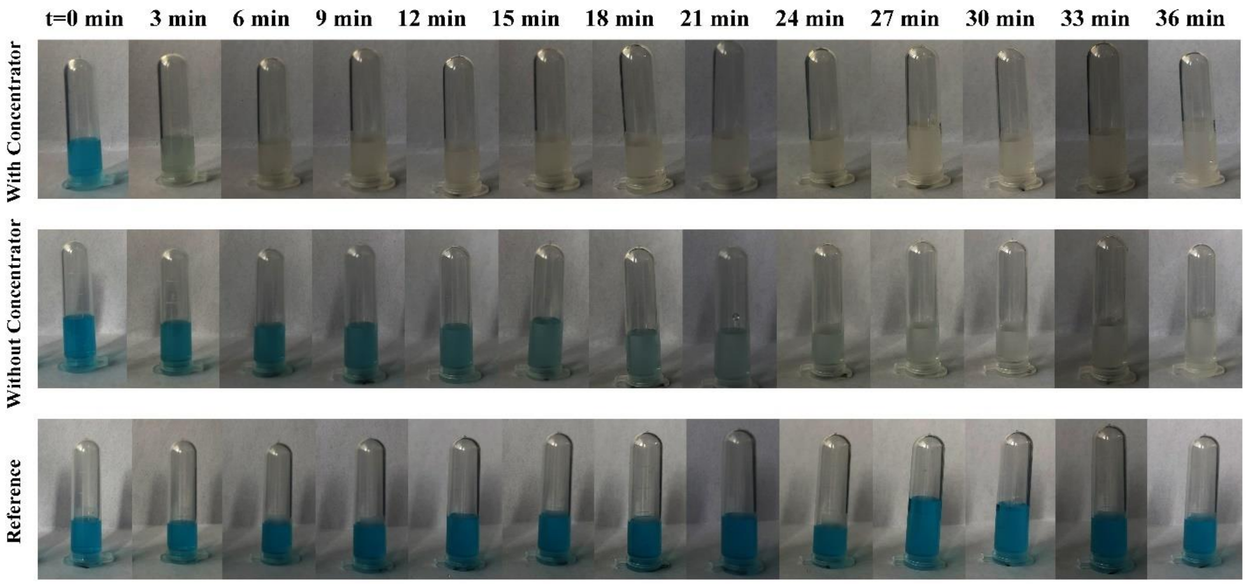

Figure S6. Experimental demonstration of catalytic efficiency of organic dye degradation. Mix the precursor solution with solution taken from inside the cloak and outside the cloak. Take 2 ml solution every 3 min to observe degrade efficiency. The last row is blank reference.

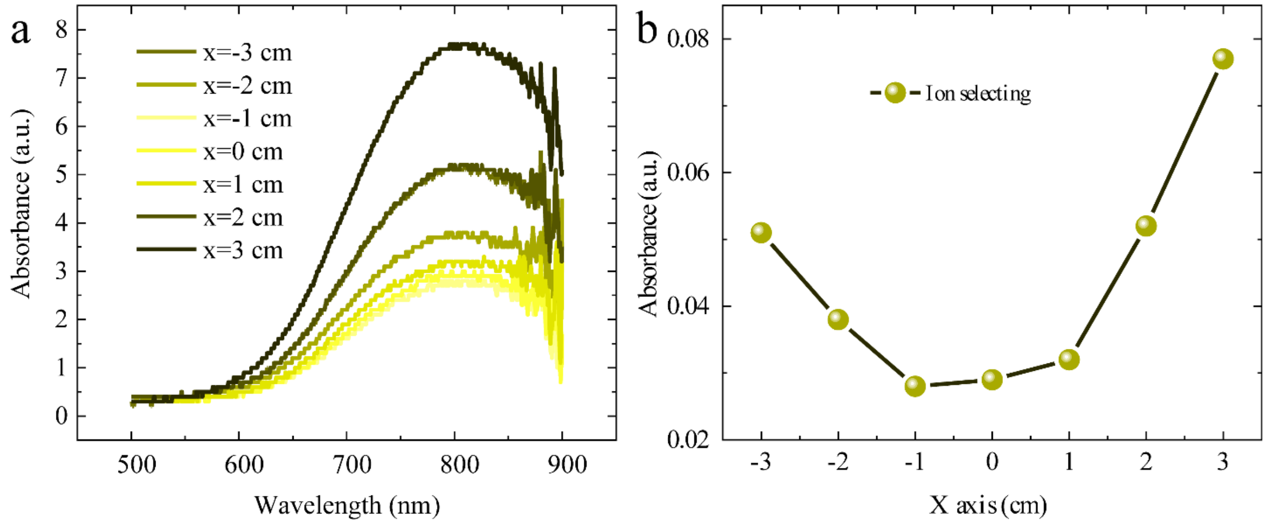

Figure S7. Measured results at line y= -3.5 cm for ion selecting metamaterials. a Measure results of absorbances of 7 spots spaced 1cm along line y= -3.5 cm. **b** Measure results of concentration distribution at line y= -3.5.
